# C‐Reactive Protein Kinetics as Prognostic Biomarkers in Stage IV‐Melanoma Treated With Immune Checkpoint Inhibitors in the Japanese Population: A Single‐Center, Retrospective Cohort Study

**DOI:** 10.1111/1346-8138.70002

**Published:** 2025-10-07

**Authors:** Ken Horisaki, Shusuke Yoshikawa, Wataru Omata, Arata Tsutsumida, Yoshio Kiyohara

**Affiliations:** ^1^ Department of Dermatology Shizuoka Cancer Center Shizuoka Japan; ^2^ Department of Dermatology Nagoya University Graduate School of Medicine Nagoya Japan

**Keywords:** C‐reactive protein, immunotherapy, melanoma, prognosis, retrospective studies

## Abstract

Malignant melanoma is an aggressive form of skin cancer with variable outcomes in response to immunotherapy. Although immune checkpoint inhibitors have improved survival in patients with advanced melanoma, predicting which patients will benefit from treatment remains a major clinical challenge. This study aimed to evaluate the prognostic value of early changes in serum C‐reactive protein levels in patients with stage IV melanoma undergoing first‐line immune checkpoint inhibitor therapy. A retrospective cohort analysis was conducted at a single center in Japan, including 123 patients treated between 2012 and 2024. Patients were classified into four groups based on their C‐reactive protein kinetics during the first 3 months of treatment: normal, flare, responder, and non‐responder. The study assessed objective response rates, disease control rates, progression‐free survival, and overall survival across these groups. Patients in the normal and flare groups demonstrated higher response and disease control rates, along with significantly longer progression‐free and overall survival, compared with non‐responders. Multivariate analysis confirmed that C‐reactive protein kinetics was an independent predictor of progression‐free and overall survival. These findings suggest that dynamic monitoring of C‐reactive protein levels may offer a practical and cost‐effective tool for early prediction of treatment outcomes in advanced melanoma. The classification system based on early inflammatory responses reflects underlying tumor–immune interactions and may help guide timely clinical decisions. This study is the first to validate this approach in a Japanese population, highlighting the relevance of inflammatory biomarkers in personalizing immunotherapy strategies for melanoma. Further prospective studies in diverse populations are warranted to confirm the clinical utility of C‐reactive protein kinetics in routine practice.

## 1 Introduction

Malignant melanoma (MM) is a highly aggressive skin cancer with a historically poor prognosis. However, the advent of immune checkpoint inhibitors (ICIs) has transformed the treatment landscape of MM [1, 2]. By blocking inhibitory signals on T cells (such as PD‐1 and CTLA‐4), ICIs unleash the immune system's antitumor response, producing remarkable, and durable clinical benefits in many patients with advanced MM. Despite this progress, the efficacy of ICI therapy is highly variable, and a substantial proportion of patients either fail to respond or eventually develop resistance. This unpredictable efficacy presents a major clinical challenge, as non‐responders may experience unnecessary toxicity and delayed access to potentially more effective alternatives [3].

To address this issue, there is an urgent need for reliable biomarkers that can predict ICI response early in the treatment course. Such biomarkers would allow clinicians to stratify patients more effectively, guiding personalized treatment strategies and enabling timely therapeutic adjustments.

Recent studies have highlighted the potential of C‐reactive protein (CRP) kinetics as a promising predictive biomarker for ICI efficacy [4, 5]. Dynamic changes in CRP levels after ICI initiation are thought to reflect the systemic inflammatory response triggered by activated antitumor immunity. Fukuda et al. [4] first described a CRP “flare” phenomenon in patients with metastatic renal cell carcinoma (mRCC), where a transient rise in CRP following ICI administration was associated with a favorable prognosis. Subsequent research has validated the predictive value of CRP kinetics in other solid tumors [6, 7, 8, 9]. These studies adopted the classification method proposed by Fukuda et al., which divided patients into three groups: flare, responder, and non‐responder. Later, Barth et al. refined this system by adding a “normal group” for patients with consistently low CRP levels. This four‐category model demonstrated improved discriminatory power for predicting treatment response and survival outcomes [5].

Nevertheless, the predictive value of CRP kinetics in MM—particularly within specific populations—remains unclear. Although several previous studies were conducted in Western populations [5, 10, 11], it has been found that the efficacy of ICI therapy for MM varies by racial group [12], necessitating the evaluation of biomarkers for each racial group. In addition, these analyses have included first‐line and later‐line ICI treatments, potentially obscuring the predictive value of CRP kinetics in the first‐line setting. Because the tumor microenvironment and patient characteristics may differ considerably between treatment lines, it is essential to evaluate biomarkers specifically in the first‐line setting to establish their clinical utility.

This study aims to determine whether early CRP kinetics can serve as a predictive biomarker for the efficacy and prognosis of first‐line ICI treatment in Japanese patients with advanced MM. Using Barth et al.'s classification system [5], we retrospectively analyzed the association between CRP kinetics and key clinical outcomes. Our goal is to assess whether evaluating CRP dynamics within the first 3 months of ICI therapy provides a valuable tool for predicting treatment outcomes in this population.

## 2 Materials and Methods

### 2.1 Study Population and Data Collection

We analyzed patients with stage IV MM treated with ICIs as first‐line systemic therapy at Shizuoka Cancer Center, Japan, between February 2012 and July 2024. The inclusion criteria were: (i) pathologically confirmed MM; (ii) primary sites including skin, mucosa, uvea, and unknown primary sites; (iii) stage IV classification according to the American Joint Committee on Cancer (AJCC) 8th edition [13]; (iv) treatment with one of the following ICIs as first‐line systemic therapy: nivolumab, pembrolizumab, or nivolumab plus ipilimumab combination therapy; and (v) availability of CRP levels before and after ICI administration. Patients with an infection that significantly affected CRP within 3 months of starting ICI were excluded. Clinical data collected included age, sex, Eastern Cooperative Oncology Group performance status (ECOG‐PS), primary tumor location, history of surgery and adjuvant therapy, stage IV details, and BRAF mutation status. For unclassified genital, anal, and urinary sites, cutaneous melanoma criteria were applied. Serum CRP levels were routinely measured at baseline (within 1 week before starting ICI therapy), at 1–2, 2–3, 4, 6, and 12 weeks after the initiation of ICI. Additional measurements were performed as clinically indicated. CRP was measured using a standard laboratory assay at Shizuoka Cancer Center.

### 2.2 Definition of CRP Kinetics

Following Barth et al. [5], patients were classified into four CRP‐response groups based on early CRP kinetics during the first 3 months after ICI initiation. The normal group included patients whose CRP never exceeded 0.5 mg/dL during the 3 months after ICI initiation. The flare group included patients whose CRP levels rose to at least twice the baseline value within 1 month of ICI initiation and then decreased below the baseline within the following 2 months. The responder group included patients who experienced a CRP decrease of at least 30% from baseline within 3 months. All remaining patients were classified as non‐responders. Patients who died prior to the completion of the 3‐month follow‐up period were categorized into a CRP group based on all available data points up to the time of death. This approach was taken to ensure the study's findings reflect real‐world clinical outcomes, including those of patients with rapid disease progression.

### 2.3 Efficacy Assessment

The primary outcomes were objective response rate (ORR), disease control rate (DCR), progression‐free survival (PFS), and overall survival (OS). Treatment response was evaluated using Response Evaluation Criteria in Solid Tumors (RECIST), version 1.1 [14]. ORR was defined as the proportion of patients achieving a complete or partial response (CR or PR, respectively). DCR was defined as the proportion achieving CR, PR, or stable disease (SD). PFS was defined as the time from ICI initiation to radiological or clinical tumor progression, final follow‐up, or death from any cause. OS was defined as the time from ICI initiation to death from any cause or final follow‐up.

### 2.4 Statistical Analysis

Baseline characteristics were compared using the Mann–Whitney *U* test for continuous variables and Fisher's exact test or the chi‐squared test for categorical variables. Fisher's exact test and the chi‐squared test were also applied to ORR and DCR analyses. OS and PFS were estimated using the Kaplan–Meier method, and differences were assessed with log‐rank tests. Cox regression analysis was used to calculate hazard ratios (HRs) for OS and PFS. For the subgroup analysis, patients were stratified by their treatment regimen: nivolumab or pembrolizumab monotherapy and nivolumab and ipilimumab combination therapy. Kaplan–Meier analysis was performed to compare the PFS and OS among the four CRP kinetic subgroups within each treatment cohort. *p* values < 0.05 were considered statistically significant. All analyses were performed using EZR (Saitama Medical Center, Jichi Medical University, Saitama, Japan), a graphical interface for R (The R Foundation for Statistical Computing, Vienna, Austria).

### 2.5 Ethics Statement

This retrospective cohort study was conducted at Shizuoka Cancer Center, Shizuoka, Japan, and was approved by the Institutional Review Board (approval number: J2025‐24). The requirement for informed consent was waived by the IRB because of the retrospective observational design. All personal data were handled in strict accordance with the ethical principles of the 1964 Declaration of Helsinki.

## 3 Results

### 3.1 Baseline Patient Characteristics

The baseline characteristics of the study population are summarized in Table 1. A total of 123 patients with stage IV MM were enrolled. Forty‐five patients (36.6%) were in the normal group, 25 (20.3%) in the flare group, 30 (24.4%) in the responder group, and 23 (18.7%) in the non‐responder group. All patients in this cohort survived for at least 4 weeks following ICI initiation. Among the 14 patients who died within 3 months, 9 were classified as non‐responders and 5 as responders. The median follow‐up period was 19.5 months in the normal group, 14.9 months in the flare group, 10.1 months in the responder group, and 5.1 months in the non‐responder group. Overall, the median age was 67 years, and 70 patients (56.9%) were men. The most common primary sites were mucosal (*n* = 44, 35.8%), cutaneous (*n* = 43, 35.0%), and acral (*n* = 20, 16.3%). More than half of the patients were BRAF mutation‐negative (*n* = 74, 60.2%) and received PD‐1 monotherapy as first‐line treatment (*n* = 90, 73.2%).

**TABLE 1:** Characteristics of patients with stage IV malignant melanoma.

Characteristic	Patient group (%)					*p*
	Total	Normal	Flare	Responder	Non‐responder	
Patients, *n* (%)	123 (100)	45 (36.6)	25 (20.3)	30 (24.4)	23 (18.7)	
Age, years						
Median [range]	67.0 [27.0, 87.0]	69.0 [28.0, 87.0]	64.0 [27.0, 86.0]	67.0 [44.0, 84.0]	67.0 [50.0, 86.0]	0.908
Sex, *n* (%)						
Male	70 (56.9)	22 (48.9)	16 (64.0)	16 (53.3)	16 (69.6)	0.341
Female	53 (43.1)	23 (51.1)	9 (36.0)	14 (46.7)	7 (30.4)	
ECOG‐PS score						
0–1	115 (93.5)	44 (97.8)	25 (100.0)	25 (83.3)	21 (91.3)	**0.039**
≥ 2	8 (6.5)	1 (2.2)	0 (0.0)	5 (16.7)	2 (8.7)	
Primary site						
Cutaneous	43 (35.0)	16 (35.6)	9 (36.0)	8 (26.7)	10 (43.5)	0.408
Acral	20 (16.3)	5 (11.1)	2 (8.0)	9 (30.0)	4 (17.4)	
Mucosal	44 (35.8)	17 (37.8)	11 (44.0)	10 (33.3)	6 (26.1)	
Uveal	8 (6.5)	5 (11.1)	2 (8.0)	0 (0.0)	1 (4.3)	
Unknown	8 (6.5)	2 (4.4)	1 (4.0)	3 (10.0)	2 (8.7)	
Primary site surgery						
Yes	85 (69.1)	39 (86.7)	12 (48.0)	19 (63.3)	15 (65.2)	**0.006**
Adjuvant therapy						
Yes	31 (25.2)	7 (15.6)	9 (36.0)	5 (16.7)	10 (43.5)	**0.029**
Details of stage IV						
M1a	7 (5.7)	5 (11.1)	1 (4.0)	1 (3.3)	0 (0.0)	0.059
M1b	23 (18.7)	13 (28.9)	4 (16.0)	3 (10.0)	3 (13.0)	
M1c	81 (65.9)	20 (44.4)	19 (76.0)	23 (76.7)	19 (82.6)	
M1d	12 (9.8)	7 (15.6)	1 (4.0)	3 (10.0)	1 (4.3)	
*BRAF*						
Mutant	19 (15.4)	8 (17.8)	4 (16.0)	4 (13.3)	3 (13.0)	0.908
Wild	74 (60.2)	29 (64.4)	15 (60.0)	17 (56.7)	13 (56.5)	
Not investigated	30 (24.4)	8 (17.8)	6 (24.0)	9 (30.0)	7 (30.4)	
First‐line treatment						
NIVO+IPI	33 (26.8)	6 (13.3)	10 (40.0)	8 (26.7)	9 (39.1)	**0.043**
PD‐1 monotherapy	33 (26.8)	39 (86.7)	15 (60.0)	22 (73.3)	14 (60.9)	
Outcome						
Dead	90 (73.2)	29 (64.4)	20 (80.0)	23 (76.7)	18 (78.3)	0.419
Alive	33 (26.8)	16 (35.6)	5 (20.0)	7 (23.3)	5 (21.7)	

*Note:* Bold letters indicate statistically significant differences: *p* < 0.05.

Abbreviations: BRAF/MEK, B‐rapidly accelerated fibrosarcoma and mitogen‐activated protein kinase; ECOG‐PS, East‐ern Cooperative Oncology Group Performance Status; NIVO+IPI, nivolumab+ipilimumab; PD‐1, anti‐PD‐1 antibody.

### 3.2 CRP Kinetics and Clinical Response

**FIGURE 1:**
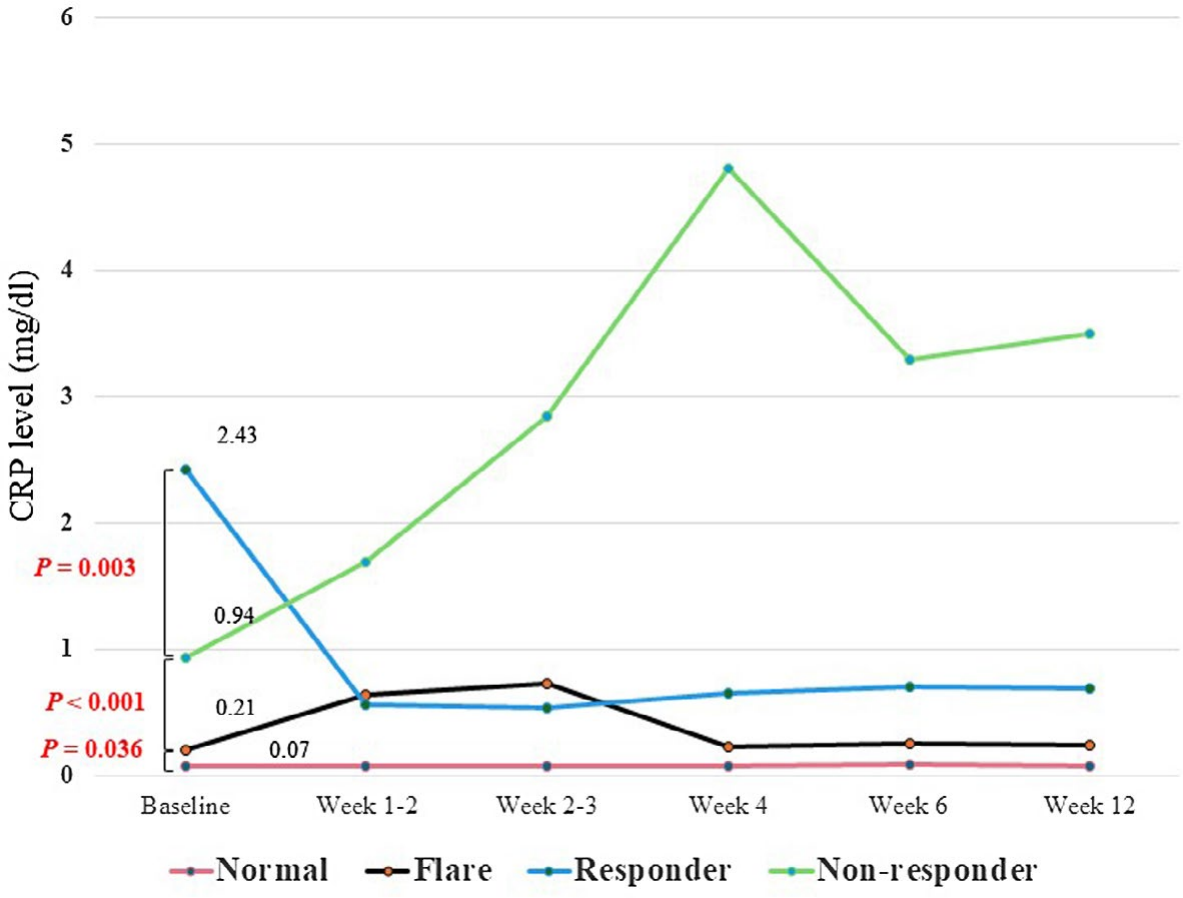
Changes in serum CRP levels after ICI administration among four CRP kinetics subgroups in malignant melanoma. The graph shows the changes in mean CRP levels for each CRP kinetics subgroup at baseline, 1–2, 2–3, 4, 6, and 12 weeks after the start of ICIs. CRP, C‐reactive protein; ICI, immune checkpoint inhibitor.

**FIGURE 2:**
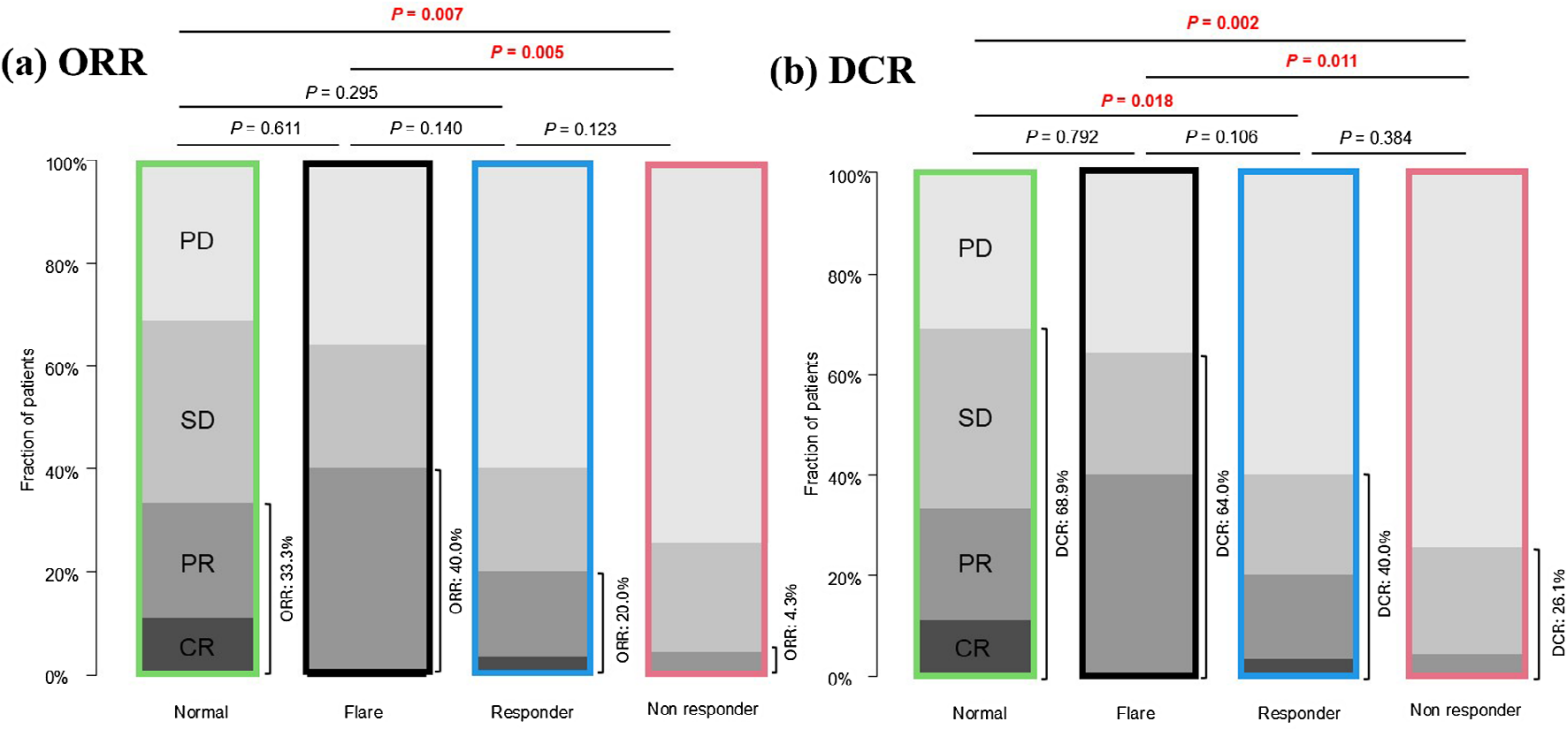
Distribution of best overall response based on RECIST in the four CRP kinetic subgroups in malignant melanoma. (a) Comparison of the objective response rate in each group. (b) Comparison of the disease control rate in each group. CR, complete response; CRP, C‐reactive protein; PD, progressive disease; PR, partial response; RECIST, response evaluation criteria in solid tumors; SD, stable disease.

CRP kinetics before and after ICI treatment are shown in Figure 1. Baseline CRP levels declined significantly in the responder, non‐responder, flare, and normal groups. In this cohort, the overall ORR was 26.0%, and the overall DCR was 52.8% (CR, 4.9%; PR, 21.1%; SD, 26.8%) (Table 2). ORR and DCR were favorable in the normal and flare groups, whereas ORR and DCR were significantly lower in the non‐responder group compared with both groups (Figure 2a,b).

**TABLE 2:** Overall response by CRP kinetics in patients with malignant melanoma.

	Patient group (%)					*p*
	Total *n* = 123	Normal *n* = 45	Flare *n* = 25	Responder *n* = 30	Non‐responder *n* = 23	
Best overall response						
Complete response	6 (4.9)	5 (11.1)	0 (0.0)	1 (3.3)	0 (0.0)	**0.005**
Partial response	26 (21.1)	10 (22.2)	10 (40.0)	5 (16.7)	1 (4.3)	
Stable disease	33 (26.8)	16 (35.6)	6 (24.0)	6 (20.0)	5 (21.8)	
Progressive disease	58 (47.2)	14 (31.1)	9 (36.0)	18 (60.0)	17 (73.9)	

*Note:* Bold letters indicate statistically significant differences: *p* < 0.05.

Abbreviation: CRP, C‐reactive protein.

### 3.3 PFS and OS

**FIGURE 3:**
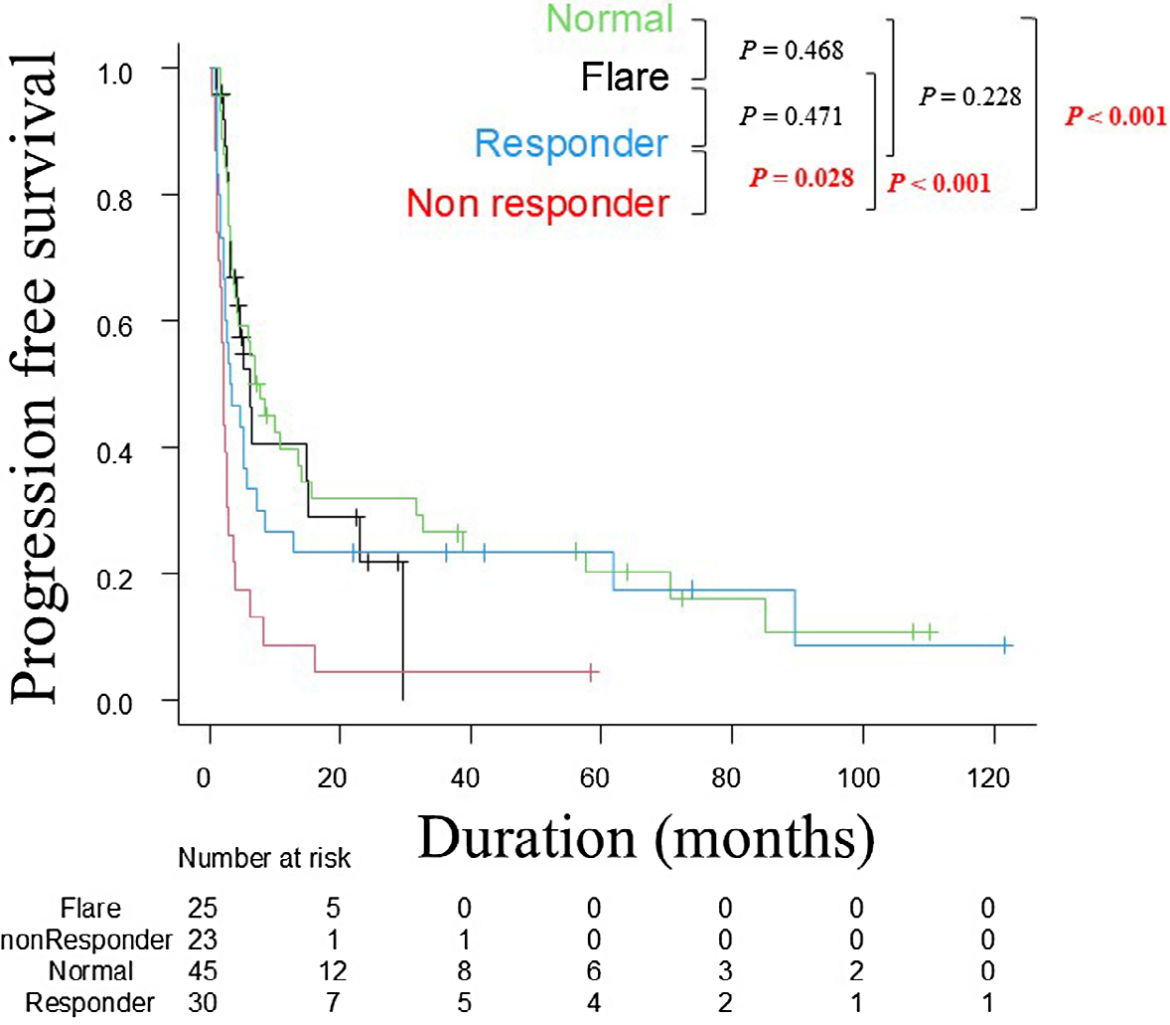
Kaplan–Meier analysis of progression‐free survival. Kaplan–Meier analysis of progression‐free survival in the four CRP kinetic subgroups. CRP, C‐reactive protein.

**FIGURE 4:**
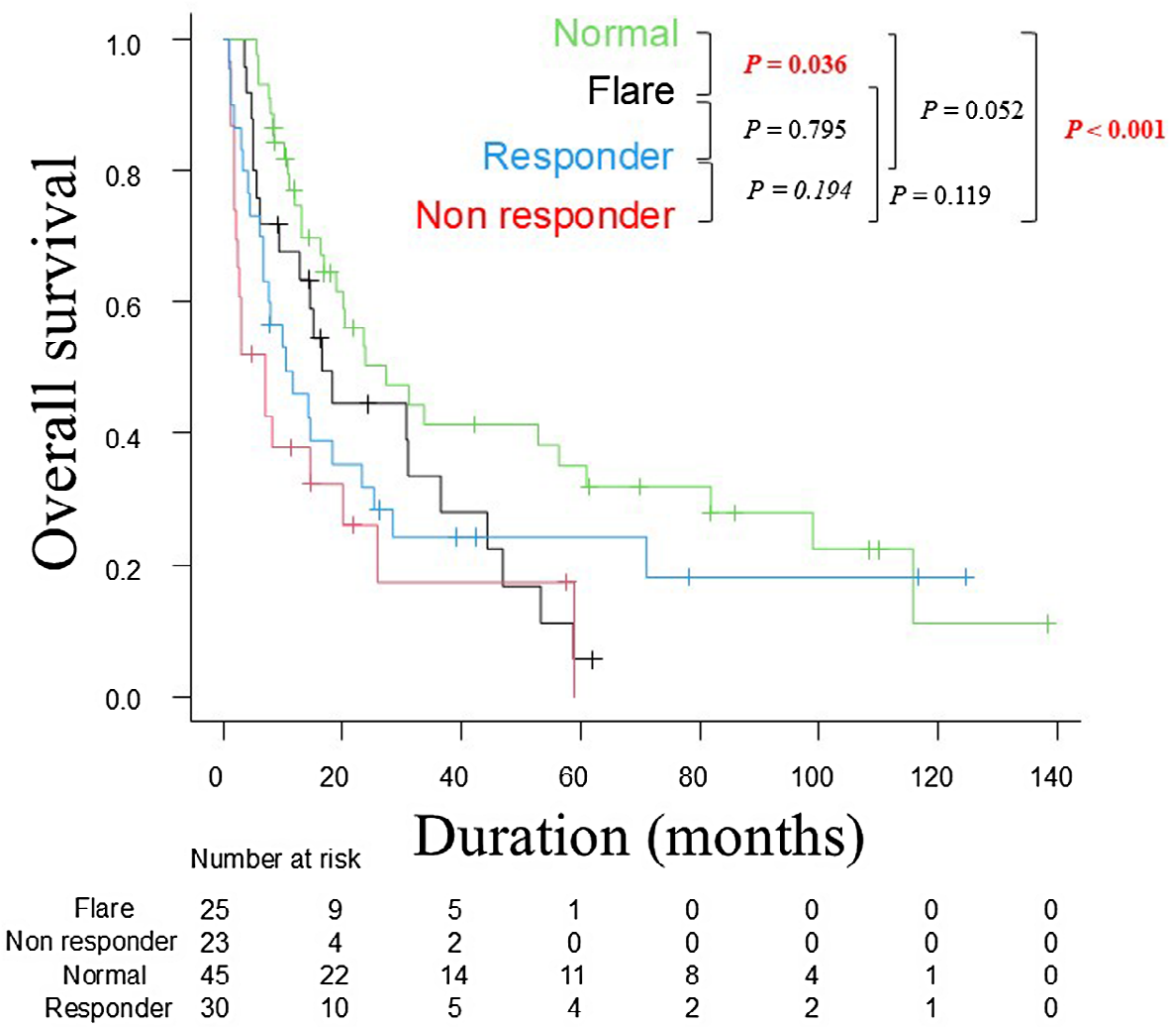
Kaplan–Meier analysis of overall survival. Kaplan–Meier analysis of overall survival in the four CRP kinetic subgroups. CRP, C‐reactive protein.

Kaplan–Meier curves for PFS in each CRP subgroup are shown in Figure 3. The median PFS was 7.6 months in the normal group, 6.3 months in the flare group, 3.3 months in the responder group, and 2.1 months in the non‐responder group. The non‐responder group had significantly worse PFS than the normal group (*p* < 0.001), flare group (*p* < 0.001), and responder group (*p* = 0.028). Kaplan–Meier curves for OS are shown in Figure 4. The median OS was 27.3 months in the normal group, 16.6 months in the flare group, 10.4 months in the responder group, and 7.0 months in the non‐responder group. For several years after ICI initiation, prognosis was best in the normal group, followed by the flare, responder, and non‐responder groups.

To address the potential confounding effect of different treatment regimens, we performed a subgroup analysis for patients receiving nivolumab or pembrolizumab monotherapy and nivolumab and ipilimumab combination therapy. In the monotherapy subgroup, consistent with the overall cohort, the non‐responder group showed significantly shorter PFS (median, 2.1 months) compared to the other three groups (*p* < 0.001, Figure S1a). The normal group had the longest OS (median, 33.8 months, Figure S1b).

In contrast, the CTLA‐4 combination therapy subgroup revealed different trends. The flare group showed a longer median PFS (6.3 months) compared to the other groups (median, 2.1–2.4 months, Figure S2a). Furthermore, the flare and non‐responder groups showed a better median OS (15.2 and 14.7 months, respectively) than the normal and responder groups (10.6 and 7.2 months, respectively, Figure S2b).

### 3.4 Univariate and Multivariate Analysis of Potential Prognostic Factors for PFS and OS

In the univariate analysis, only ECOG‐PS (≥ 2; HR, 2.578; 95% CI, 1.117–5.948; *p* = 0.026) and CRP kinetics (normal: HR, 0.328; 95% CI, 0.190–0.565; *p* < 0.001; flare: HR, 0.367; 95% CI, 0.194–0.695; *p* = 0.002; responder: HR, 0.448; 95% CI, 0.250–0.803; *p* = 0.007) were significantly associated with PFS (Table 3). For OS, ECOG‐PS (≥ 2; HR, 2.312; 95% CI, 1.066–5.014; *p* = 0.034) and CRP kinetics (normal: HR, 0.340; 95% CI, 0.187–0.620; *p* < 0.001; flare: HR, 0.575; 95% CI, 0.303–1.090; *p* = 0.090; responder: HR, 0.586; 95% CI, 0.314–1.095; *p* = 0.094) were also significantly associated (Table 4). In the multivariate analysis, only CRP kinetics remained independent prognostic factors for PFS (normal: HR, 0.369; 95% CI, 0.204–0.668; *p* < 0.001; flare: HR, 0.384; 95% CI, 0.201–0.733; *p* = 0.003; responder: HR, 0.473; 95% CI, 0.255–0.880; *p* = 0.018) (Table 3). For OS, CRP kinetics were also the only independent prognostic factors (normal: HR, 0.394; 95% CI, 0.208–0.746; *p* = 0.004; flare: HR, 0.615; 95% CI, 0.323–1.168; *p* = 0.137; responder: HR, 0.597; 95% CI, 0.305–1.167; *p* = 0.132) (Table 4).

**TABLE 3:** Univariate and multivariate analysis of potential prognostic factors for progression‐free survival.

	Univariate analysis			Multivariate analysis		
	Hazard ratio	95% CI	*p*	Hazard ratio	95% CI	*p*
CRP kinetics						
Non‐responder	Reference			Reference		
Normal	0.328	0.190–0.565	**< 0.001**	0.369	0.204–0.668	**< 0.001**
Flare	0.367	0.194–0.695	**0.002**	0.384	0.201–0.733	**0.003**
Responder	0.448	0.250–0.803	**0.007**	0.473	0.255–0.880	**0.018**
Age	1.004	0.988–1.021	0.625	1.010	0.992–1.028	0.286
Sex						
Male	Reference			Reference		
Female	0.757	0.495–1.159	0.201	0.678	0.445–1.034	0.071
ECOG‐PS						
Score 0–1	Reference			Reference		
Score ≥ 2	2.578	1.117–5.948	**0.026**	1.874	0.810–4.337	0.142
Primary site						
Acral	Reference					
Cutaneous (non‐acral)	1.043	0.562–1.934	0.895			
Mucosal	0.682	0.366–1.272	0.229			
Uveal	0.811	0.313–2.102	0.666			
Unknown primary	1.351	0.567–3.216	0.497			
Stage						
M1a	Reference					
M1b	1.201	0.401–3.598	0.743			
M1c	1.712	0.622–4.716	0.298			
M1d	2.045	0.627–6.667	0.235			
BFAF						
Wild‐type	Reference					
Mutant	0.951	0.527–1.716	0.867			
Primary site surgery						
No	Reference					
Yes	0.774	0.498–1.201	0.253			
Adjuvant therapy						
No	Reference					
Yes	1.104	0.681–1.792	0.688			
First‐line treatment						
NIVO+IPI	Reference					
PD‐1 monotherapy	0.645	0.408–1.018	0.060	0.802	0.475–1.356	0.411

*Note:* Bold letters indicate statistically significant differences: *p* < 0.05.

Abbreviations: BRAF/MEK, B‐rapidly accelerated fibrosarcoma and mitogen‐activated protein kinase; ECOG‐PS, East‐ern Cooperative Oncology Group Performance Status; NIVO+IPI, nivolumab+ipilimumab; PD‐1, anti‐PD‐1 antibody.

**TABLE 4:** Univariate and multivariate analysis of potential prognostic factors for overall survival.

	Univariate analysis			Multivariate analysis		
	Hazard ratio	95% CI	*p*	Hazard ratio	95% CI	*p*
CRP kinetics						
Non‐responder	Reference			Reference		
Normal	0.340	0.187–0.620	**< 0.001**	0.394	0.208–0.746	**0.004**
Flare	0.575	0.303–1.090	0.090	0.615	0.323–1.168	0.137
Responder	0.586	0.314–1.095	0.094	0.597	0.305–1.167	0.132
Age	1.006	0.991–1.022	0.426	1.012	0.992–1.032	0.261
Sex						
Male	Reference			Reference		
Female	0.675	0.451–1.009	0.056	0.783	0.498–1.230	0.288
ECOG‐PS						
Score 0–1	Reference			Reference		
Score ≥ 2	2.312	1.066–5.014	**0.034**	2.333	0.926–5.880	0.072
Primary site						
Acral	Reference					
Cutaneous (non‐acral)	1.608	0.889–2.908	0.116			
Mucosal	0.821	0.450–1.497	0.520			
Uveal	0.949	0.389–2.314	0.909			
Unknown primary	1.654	0.705–3.880	0.247			
Stage						
M1a	Reference					
M1b	0.709	0.262–1.915	0.497			
M1c	0.966	0.387–2.410	0.941			
M1d	1.079	0.366–3.180	0.891			
BFAF						
Wild‐type	Reference					
Mutant	1.475	0.845–2.573	0.172			
Primary site surgery						
No	Reference					
Yes	0.851	0.555–1.304	0.459			
Adjuvant therapy						
No	Reference					
Yes	1.025	0.646–1.626	0.918			
First‐line treatment						
NIVO+IPI	Reference					
PD‐1 monotherapy	0.729	0.461–1.152	0.175	0.673	0.392–1.157	0.152

*Note:* Bold letters indicate statistically significant differences: *p* < 0.05.

Abbreviations: BRAF/MEK, B‐rapidly accelerated fibrosarcoma and mitogen‐activated protein kinase; ECOG‐PS, Eastern Cooperative Oncology Group Performance Status; NIVO + IPI, nivolumab + ipilimumab; PD‐1, anti‐PD‐1 antibody.

## 4 Discussion

In this study, we examined whether CRP kinetics within 3 months of ICI initiation could predict treatment efficacy in Japanese patients with MM. The normal and flare groups were more likely to benefit from ICI therapy, whereas the non‐responder group had a significantly worse prognosis. These findings suggest that the evaluation of CRP kinetics may be useful as a predictive biomarker of ICI efficacy in Japanese patients with MM.

Fukuda et al. [4] first described the CRP flare phenomenon, in which CRP levels initially rise after ICI initiation and subsequently fall below baseline. They analyzed early CRP dynamics in 42 patients with mRCC treated with nivolumab as second‐line or later therapy, classifying patients as flare, responder, or non‐responder. Their analysis showed that flare and responder groups had higher ORR and longer survival compared with non‐responders. Subsequent studies confirmed similar favorable prognostic results in mRCC, metastatic urothelial carcinoma, and advanced non‐small cell lung cancer [6, 7, 8, 9]. Barth et al. [5], however, noted that Fukuda's method inadvertently included patients with consistently low CRP (normal prognosis) in the non‐responder group. They proposed a four‐category model by adding a normal group. In two Austrian cohorts (562 and 474 patients with solid malignancies), this model improved prediction of response rates, disease progression, and survival. In a sub‐analysis of MM, normal and flare groups had significantly better PFS (normal, HR, 0.39; 95% CI, 0.27–0.56; flare, HR, 0.37; 95% CI, 0.27–0.65), and OS (normal, HR, 0.30; 95% CI, 0.15–0.64; flare, HR, 0.34; 95% CI, 0.12–0.98) than non‐responders.

Regarding MM, a retrospective German study applied Fukuda's classification to 87 patients treated with ICIs. Median PFS was 0.7, 0.6, and 0.2 years in the flare, responder, and non‐responder groups (*p* = 0.017), whereas median OS was 2.2, 1.5, and 1.0 years, respectively (*p* = 0.014) [10]. More recently, a Turkish cohort study of 104 patients with MM used Barth's four groups' classification [11]. The median PFS was 33.57 months in the normal group, 8.83 months in the flare group, 10.90 months in the responder group, and 4.80 months in the non‐responder group (*p* < 0.001); the median OS was 54.5, 21.5, 38.1, and 11.9 months, respectively (*p* < 0.001). Compared with these findings, our study showed a similar trend but with overall shorter PFS and OS. This may reflect racial differences in ICIs outcomes [15]. Notably, East Asians have higher rates of acral and mucosal melanomas [16, 17, 18, 19, 20], which respond less favorably to ICIs, as well as lower tumor mutation burden in cutaneous melanoma [21, 22, 23]. Unlike prior MM studies, our work is the first in an East Asian population and focuses exclusively on first‐line ICI treatment. Because outcomes vary by line of therapy, treatment regimen, race, and subtype, further evaluation of CRP kinetics across patient subgroups is warranted. Subgroup analyses performed to examine the impact of treatment regimens revealed distinct patterns of CRP dynamics and their prognostic significance depending on the treatment regimen. In the nivolumab or pembrolizumab monotherapy group, our findings were consistent with the overall cohort, reinforcing the robustness of CRP kinetics as a prognostic marker. Notably, the combination therapy group presented a different outcome. The superior prognosis observed in the flare group suggests that the CRP flare in this context might represent a more intense and effective anti‐tumor immune activation, rather than a mere inflammatory response. CTLA‐4 inhibition is known to induce broader T‐cell activation and proliferation, which may lead to a different inflammatory profile compared to PD‐1 monotherapy [24]. However, this observation must be interpreted with caution owing to the small sample size of the combination therapy subgroup. Furthermore, our analysis revealed that the treatment regimen itself did not emerge as an independent prognostic factor. This suggests that while different treatments may induce distinct inflammatory profiles, it is the individual patient's underlying immune response, as captured by early CRP kinetics, that appears to be a more critical determinant of long‐term outcomes than the specific therapy they received. Taken together with prior studies, our findings support CRP kinetics as a broadly useful prognostic indicator in MM patients receiving ICIs.

The relationship between CRP kinetics and antitumor immunity provides biological insight into our findings. CRP is not only a systemic inflammation marker but is also tightly linked to tumor immunity [25, 26]. A CRP flare likely reflects a strong immune response, as ICI‐activated T cells release cytokines such as IL‐6, which stimulate hepatic CRP production [4]. Thus, patients in the flare group, who demonstrated this dynamic response, derived greater benefit and had better prognoses. Conversely, chronic inflammation creates an immunosuppressive tumor microenvironment enriched with M2 macrophages and regulatory T cells, impairing antitumor immunity [27]. This may explain why responder and non‐responder groups with high baseline CRP levels had weaker immune activation than the flare group. In patients with solid tumors treated with ICI, a high baseline CRP has also been reported as a poor prognostic factor [28, 29]. Nonetheless, responders fared better than non‐responders, suggesting that in responders, ICIs successfully overcame the suppressive microenvironment and shifted inflammation toward an antitumor profile. Thus, group differences appear to reflect not only the amount but also the quality of inflammation and tumor responsiveness. The favorable prognosis in the normal group may relate to low baseline inflammation and the inclusion of patients who fit flare or responder patterns but whose CRP levels never exceeded 0.5 mg/dL [5].

Clinically, these findings are significant. Evaluating early CRP kinetics may help physicians predict ICI effectiveness and identify patients unlikely to benefit. For non‐responders, this information could prompt earlier consideration of alternative or combination treatments, reducing unnecessary toxicity and treatment delays. CRP measurement is simple, inexpensive, and widely available, making it a highly feasible biomarker in practice.

However, this study has limitations. First, as a single‐center retrospective study, it is prone to selection and information bias. Second, the relatively small sample size limits statistical power, and larger validation cohorts are needed. The findings from subgroup analyses, particularly for the combination therapy group, should therefore be interpreted cautiously due to the small number of subjects. Third, limited blood sampling may have missed flare or other kinetic patterns. Fourth, other inflammatory markers (e.g., cytokines) were not evaluated, which might have provided a fuller picture of the immune response. A fifth limitation is the potential bias resulting from including patients with early mortality. Patients who died within 3 months were predominantly classified as non‐responders, which may overstate survival in the normal and flare groups. However, a significant strength of this methodology is its ability to reflect real‐world outcomes and identify a critical high‐risk group early in their treatment course.

This study is the first to demonstrate that early CRP kinetics can serve as a biomarker for predicting efficacy and prognosis in Japanese patients with advanced MM receiving first‐line ICIs. The normal and flare groups showed favorable outcomes compared with non‐responders. These results suggest that CRP kinetics can be a valuable tool for predicting outcomes and guiding personalized strategies for MM patients. They also align with prior studies, supporting the role of CRP kinetics across populations and treatment settings.

## Ethics Statement

Approval of the research protocol by an Institutional Review Board: The protocol for this study was reviewed and approved by the Ethics Committee of Shizuoka Cancer Center (2025/4/25), approval no. J2025‐24.

## Consent

The requirement for informed consent was waived by the IRB because of the retrospective observational design.

## Conflicts of Interest

The authors declare no conflicts of interest.

## Supporting information


**Data S1:** jde70002‐sup‐0001‐FigureS1‐S2.docx.

## Data Availability

The data that support the findings of this study are available from the corresponding author upon reasonable request.
