# Supplementary material for: C‐Reactive Protein Kinetics as Prognostic Biomarkers in Stage IV‐Melanoma Treated With Immune Checkpoint Inhibitors in the Japanese Population: A Single‐Center, Retrospective Cohort Study
Source: J Dermatol. 2025 Oct 7;53(1):14–24. doi: 10.1111/1346-8138.70002 (PMC12784795; doi:10.1111/1346-8138.70002)
Supplement: Supplementary file 1 — Data S1: jde70002‐sup‐0001‐FigureS1‐S2.docx. [file JDE-53-14-s001.docx]

**Supplementary materials**


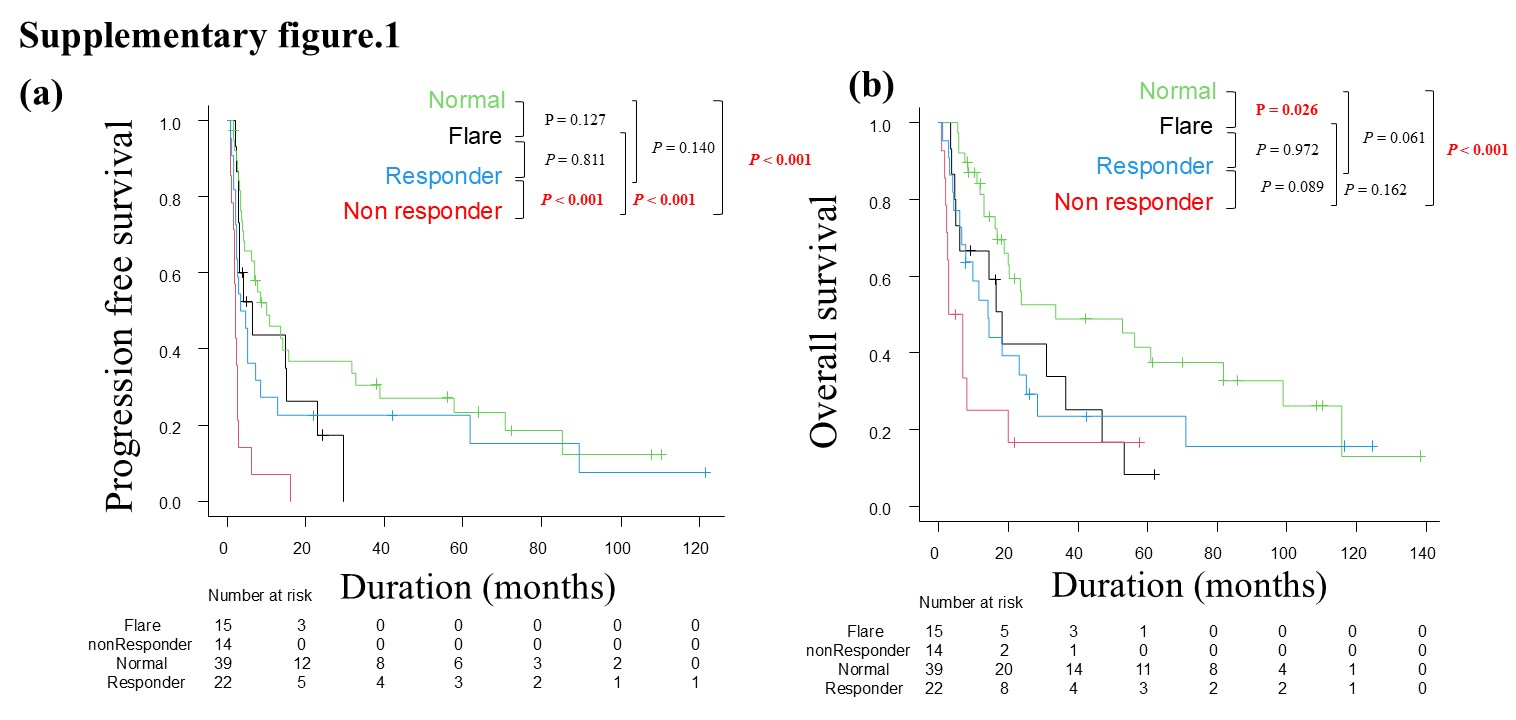
**Supplementary figure 1.** **Kaplan–Meier analysis of progression-free survival and overall survival limited to nivolumab or pembrolizumab monotherapy**

(a) Kaplan–Meier analysis of progression-free survival (PFS) in the four CRP kinetic subgroups. The median PFS was 9.9 months in the normal group, 6.4 months in the flare group, 4.0 months in the responder group, and 2.1 months in the non-responder group. The non-responder group had significantly worse PFS than the other three groups (*P* < 0.001, respectively). (b) Kaplan–Meier analysis of overall survival (OS) in the four CRP kinetic subgroups. The median OS was 33.8 months in the normal group, 18.3 months in the flare group, 14.3 months in the responder group, and 5.1 months in the non-responder group. Consistent with the overall trend, the normal group tended to have a better prognosis than the other three groups.


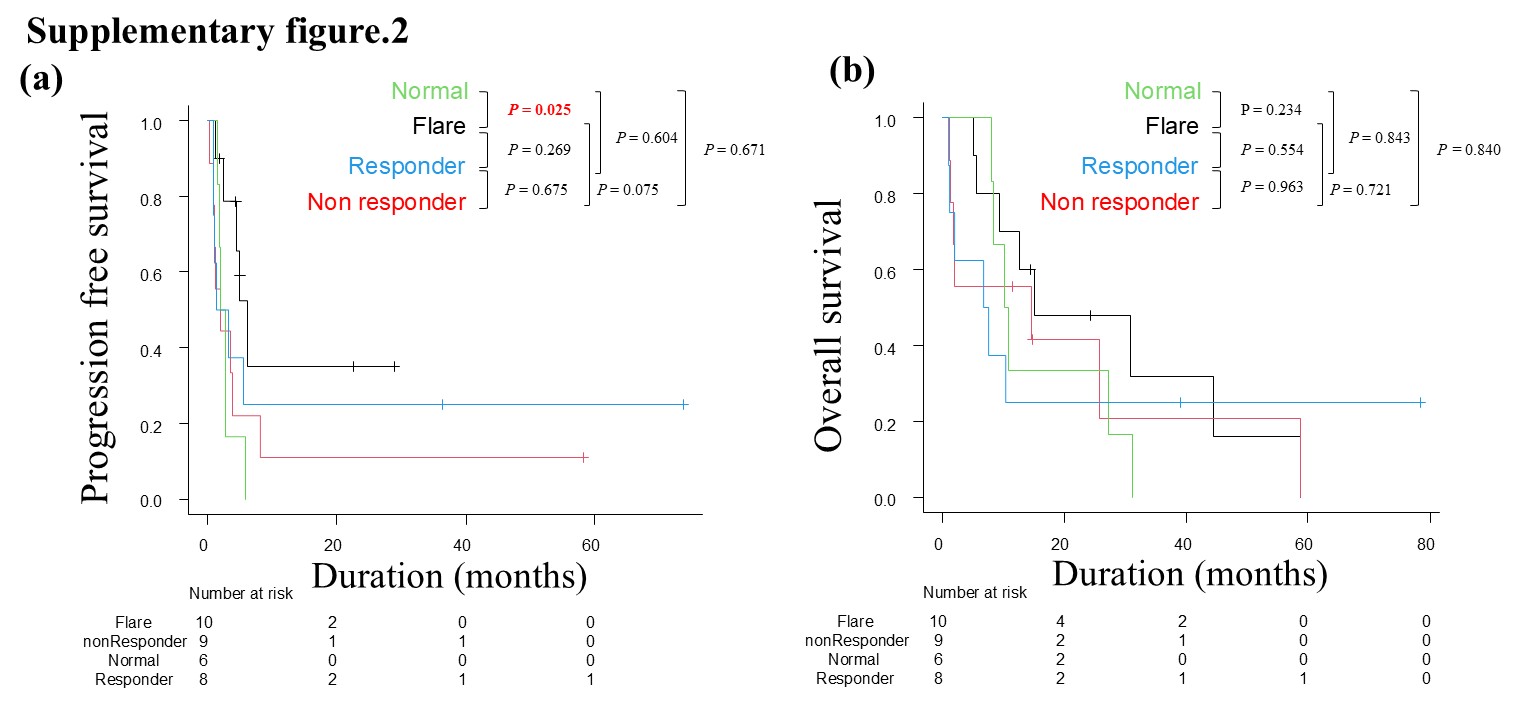
**Supplementary figure 2.** **Kaplan–Meier analysis of progression-free survival and overall survival limited to nivolumab and ipilimumab combination therapy**

(a) Kaplan–Meier analysis of progression-free survival (PFS) in the four CRP kinetic subgroups. The median PFS was 2.4 months in the normal group, 6.3 months in the flare group, 2.3 months in the responder group, and 2.1 months in the non-responder group. (b) Kaplan–Meier analysis of overall survival (OS) in the four CRP kinetic subgroups. The median OS was 10.6 months in the normal group, 15.2 months in the flare group, 7.2 months in the responder group, and 14.7 months in the non-responder group.
